# Dynamical system of a time-delayed of rigid rocking rod: analytical approximate solution

**DOI:** 10.1038/s41598-023-32743-w

**Published:** 2023-04-05

**Authors:** Galal M. Moatimid, T. S. Amer

**Affiliations:** 1 Department of Mathematics, Faculty of Education, Ain Shams University, Cairo, Egypt; 2 Department of Mathematics, Faculty of Science, Tanta University, Tanta, 31527 Egypt

**Keywords:** Mathematics and computing, Applied mathematics

## Abstract

The stability analysis of a rocking rigid rod is investigated in this paper using a time-delayed square position and velocity. The time delay is an additional safety against the nonlinearly vibrating system under consideration. Because time-delayed technologies have lately been the core of several investigations, the subject of this inquiry is extremely relevant. The Homotopy perturbation method (HPM) is modified to produce a more precise approximate outcome. Therefore, the novelty of the exciting paper arises from the coupling of the time delay and its correlation with the modified HPM. A comparison with the fourth-order Runge–Kutta (RK4) technique is employed to evaluate the precision between the analytical as well as the numerical solutions. The study allows for a comprehensive examination of the recognition of the outcome of the realistic approximation analytical methodology. For different amounts of the physical frequency and time delay factors, the time histories of the found solutions are depicted in various plots. These graphs are discussed in the context of the shown curves according to the relevant parameter values. The organized nonlinear prototype approach is examined by the multiple-time scale method up to the first approximation. The obtained results have periodic behavior and a stable manner. The current study makes it possible to carefully examine the findings arrived at by employing the analytical technique of practicable estimation. Additionally, the time delay performs as extra protection as opposed to the system potential for nonlinear oscillation.

## Introduction

The computational asymptotic methodologies of nonlinear applications seemed to be of great significance to engineers and scientists as nonlinear science advances so quickly. However, we can easily use simulation studies to find solutions of linear systems. It is still extremely challenging to solve nonlinear problems theoretically. One of the perfect examples of a Hamilton system was a Duffing oscillator. Simple generalizations of these oscillators, including cubic-quintic Duffing oscillators, haven't been thoroughly researched^1–4^. Conclusions for the impacts of the base stiffness, position, and amount of combined mass on the change of the vibration cycle graphically in non-dimensional presentations were demonstrated^1^. A brand-new factor iteration method to analyze the Duffing equation as having powerful and high-order nonlinearity was suggested^2^. It was demonstrated that the nonlinear frequency gives rise to accurate outcomes, in contrast to the linearized approach, parametrized perturbation procedure, and variational repetition procedure suggested by Prof. He. In order to arrive at analytical approximations and numerical explanations for the cubic-quintic Duffing-van der Pol oscillator, a number of methodologies, considering the prospective purposes in manufacturing, integrated circuit technology, physical processes, and natural science was employed^3^. The parametric Duffing oscillator stability evaluation in light of its numerous uses in science and engineering was examined^4^. Both resonance and non-resonance situations were examined. The perturbed solutions and the stability analysis were graphically verified by numerical approximations. Using the combination method, a uniform explanation of the quintic Duffing equation was reported^5^. This method involved developing the restoring force in Chebyshev's nonlinear differential equation and approximating it with a cubic Duffing oscillator where the constants for the linear and cubic components change on the primary amount.

In real life, there were occurrences where objects were not steadily attached to their framework but instead let rock or drop on the supporting material. These include unsafe supplies in transportation, petroleum cracking towers, air distillation columns, liquid gas tanks, and nuclear fuel cells in reactors. The most prominent example of how structures behave when they rock was certainly when they shake during earthquakes. Despite their familiarity and apparent simplicity, the rocking and overturning of stiff bodies in response to foundation stimulation pose difficult problems. The main motivation for understanding the issue has been the possible use of the rocking problem to prevent machinery, furniture, and structures from toppling over and endangering people when subjected to shaking during an earthquake. Satisfactory results of the isolation system rocking were obtained in Refs.^6,7^. Ganji et al.^8^ developed a cubic-quintic Duffing oscillator approach to approximate the performance of an inflexible rod swaying on a circular surface with no sliding. Khah and Ganji^9^ used the energy balancing approach to analyze the previous problem^8^. The new methodology showed high effectiveness and convenience and lacked the necessity for linearization or tiny perturbation. Additionally, a uniformly rigid rocking rod was analyzed^10^.

In industrial control mechanisms, time delay is common. It may become unstable or function poorly if there is a time delay, making it more difficult to accurately examine the system. In other words, time delays are a constant feature of controlled system feedback and have a significant impact on their dynamics^11^. For instance, even for very low time delays, a classical Duffing structure with delayed speed response displays an endless variety of regular movements; yet if the duration delay vanishes, the dynamics of this system are extremely straightforward. Delay feedback control, in contrast, has proven to be one of the most reliable and adaptable methods for controlling chaos in nonlinear dynamic systems^12^. Over the past few decades, controlled mechanical systems with time delays have received a lot of attention. Additionally, researchers in various disciplines, including biology, population dynamics, industrial machinery dynamics, and neural networks, have paid close attention to the dynamics of delay prototypes^13^. Through the use of a Duffing equation with delayed speed response, Wang and Hu^14^ presented research work on the viability of perturbation approaches as the multiple scales technique, and the Lindstedt–Poincaré methodology, to mention a few. Tunç^15^ provided the necessary conditions. Through building a Lyapunov function, a new finding was generated that incorporated and enhanced certain related results already found in the pertinent literature. An excited Van der Pol-Duffing oscillator nonlinearity was suppressed using time-delayed position and velocity^16^. The time delay served as an additional safeguard compared to the nonlinearly vibrating system under consideration. Technologies with a temporal delay have recently been the aim of various investigations; therefore, the current study is particularly examined.

As is commonly held, the majority of practical and technical implementations demand nonlinear equations. Perhaps, functional, differential, integral, or integro-differential equations make up these equations. These equations are quite challenging to obtain an exact solution. Subsequently, it becomes fundamental to employ numerical solutions in different directions. Regardless of the analytical approach, numerical solutions are more informative during specific time intervals. Therefore, perturbation approaches have developed in a variety of ways, from the standard strained factor approach to methods including those that depend on so many time scales. He et al.^17^ utilized the Poincaré–Lindstedt technique to arrive at a roughly restricted solution for the hybrid structure. It was discovered that the approximate solution together with the RK4 were comparable. In reality, the presence of a little parameter in the scheme under study is required for all perturbation methods. Consequently, without this parameter, the problem is somewhat restricted. The Mathematician Chinese Prof. He^18^ discovered a novel perturbation technique that was not dependent on such a little parameter. This method allows for the placement of a small artificial embedding parameter where $\delta \in [0,1]$. If, $\delta = 0$, then the zero-order differential equation needs to have an accurate solution. Moatimid^19^ and ^20^ used an extended frequency concept combined with the HPM and Laplace transform to analyze a parameterized Duffing equation to arrive at a valid constricted formula of solution. There have been recent efforts that were connected to the current manuscript^21–23^.

Given the implications of the above-mentioned features, and because of their extreme sensitivity to dynamic loading, differences in geometry, and dissipation difficulties, evaluating the rocking and overturning responses of rigid blocks to earthquakes is a challenge. This paper introduces the literature on traditional and cutting-edge rocking motion theories. This work has the goal of examining the motion behavior of rigid rocking rods with no slippage. The governing equation of motion (EOM) is regulated as an ordinary differential equation (ODE) contract with classical mechanics. The current study uses a connection between the Laplace transform and homotopy perturbation methods to identify perturbed solutions. The association of the time delay and its relationship with the improved HPM is what gives the interesting paper its uniqueness. It is possible to perform stability analysis using a nonlinear frequency. The residual of the text is constructed as follows: Finding a rough solution for nonlinear oscillation with an extended frequency is the focus of “Modification of the HPM”. “Linearized Stability” provides the linearized stability analysis. "Multiple-time scales method" describes the multiple-time scales technique. Furthermore, "Concluding remarks" presents additional understandings.

## Modification of the HPM

**Figure 1:**
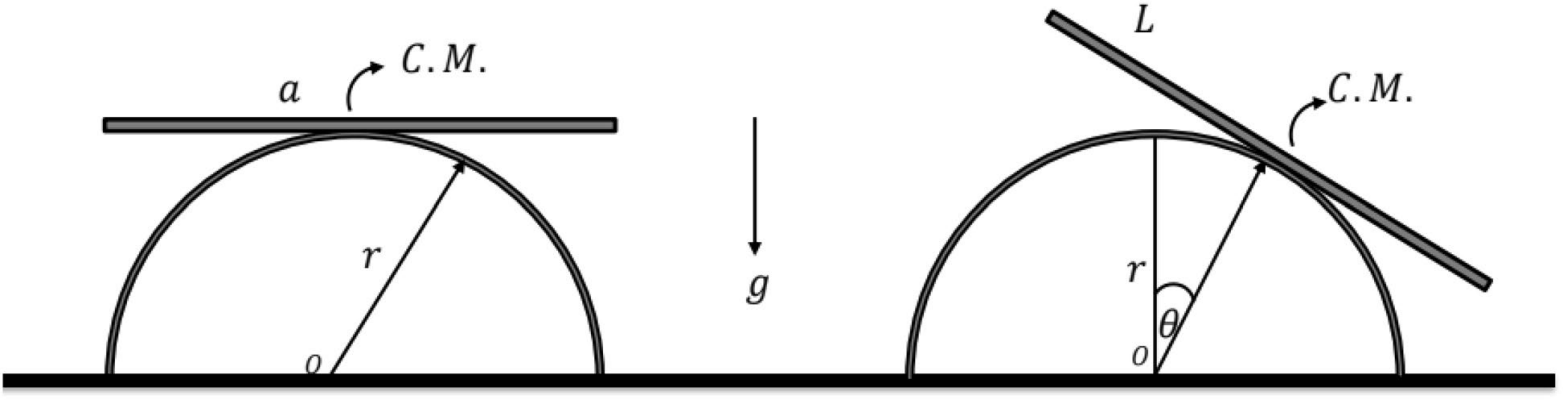
Sketches the model under consideration.

Returning to our previous paper^10^, the EOM of the uniform rigid rocking rod has been derived and taken the following form:

1 $$\frac{{Ma^{2} }}{12}\ddot{\theta } + Mr^{2} \left( {\theta^{2} \ddot{\theta } + \theta \dot{\theta }^{2} } \right) + Mrg\cos \theta = 0,$$

where a list of all the variables used in Eq. (1) is provided at the beginning of the article. The drawing of the basic prototype is depicted in Fig. 1.

Equation (1) should just be expressed in a non-dimensional form for more simplicity. There are several ways to accomplish this, mostly depending on the properties of mass, length, and time that are selected. Consider that these characteristics are chosen as: $M,\,\,r,\,\,{\text{and}}\,\,\sqrt {\text{r/g}}$ correspondingly. Additionally, the Taylor expansion is used to remove the existence of the restoring force. Subsequently, Eq. (1) will be transformed into the following simplified form:

2 $$\ddot{\theta } + \omega^{2} \theta + \omega^{2} (\theta^{2} \ddot{\theta } + \theta \dot{\theta }^{2} ) - \frac{{\omega^{2} }}{2}\theta^{3} = 0.$$

As is well known, time-delayed control was suggested to manage the nonlinear vibrations. The loop delay can significantly contribute to both stabilizing/destabilizing the structure, as was earlier shown^23^. It was compared to the previous results to show how well the applied time delay suppressed nonlinear oscillations of the structure. Nevertheless, when the ideal time delay was considered, the generated theoretical and computational inspections indicated that the nonlinear position and nonlinear velocity were the best at suppressing the vibration. Furthermore, Saeed et al.^24^ suggested a straightforward technique for designing the ideal loop delay amounts in a such way that enhances the system profile. Additionally, Saeed et al.^25^ looked at the effectiveness of time-delayed linear and nonlinear feedback controllers for position, speed, and acceleration. According to investigators, the time-delayed cubic acceleration control was the best at suppressing bifurcations and reducing vibration. Therefore, in light of the aforementioned achievement, we lay prominence on the square time delay in the position and velocity of the existing model.

From now on, the EOM becomes:

3 $$\ddot{\theta } + \omega^{2} \theta + \omega^{2} [\theta^{2} (t - \tau )\ddot{\theta } + \theta \dot{\theta }^{2} (t - \tau )] - \frac{{\omega^{2} }}{2}\theta^{3} = 0.$$

It is preferable to picture the initial conditions as follows:

4 $$\theta (0) = 0,\,\,\,\,\,\,\dot{\theta }(0) = 1.$$

As clearly explained in our previous studies^19,20,26^, the HPM can provide a wide range of approximation solutions. One of these methods leads to a conventional solution that includes secular terms, and the cancellation of these secular terms provides a trivial solution that is not required. An alternative one produces a consistently satisfactory solution by using the expanded frequency conception; unfortunately, the obtained solution does not pass through the numerical solution. It is therefore required to modify Homotopy once again. Therefore, one is able to re-examine the fundamental Homotopy equation utilizing a recent development in place of the traditional extension to look into the consequences of the delay parameter which is superior at preventing bifurcations and decreasing vibration. Subsequently, we think that $\theta (t,\rho )$ can be expanded upon in light of our earlier work^16^. The processes to get the needed solution are as follows:

The HPM is built generally on the fundamental Homotopy formula^16,26,27,28^:

5 $$\ddot{\theta } + \omega^{2} \theta + \omega^{2} \rho \left\{ {[\theta^{2} (t - \tau )\ddot{\theta } + \theta \dot{\theta }^{2} (t - \tau )] - \frac{1}{2}\theta^{3} } \right\} = 0,\,\,\,\,\,\,\,\rho \in [0,\,1].$$

6 $$\theta (t,\rho ) = e^{ - \rho \tau \,t} [\theta_{0} (t) + \rho \,\theta_{1} (t) + \rho^{2} \theta_{2} (t) + \ldots ].$$

Applying the technique of the previously comprehended examination, the established natural frequency can be increased as shown below^19,20^:

7 $$\sigma^{2} = \omega^{2} + \sum\limits_{i = 1}^{n} {\rho^{i} \sigma_{i} } .$$

One insert Eqs. (6) and (7) into the Homotopy equation to create the solution of Eq. (3). Consequently, the precise analytic solution to the zero-order problem, which corresponds to the I.C, is given by

8 $$\theta_{0} (t) = \frac{1}{\sigma }\;\sin \,\sigma t.$$

It is observed that the time delay of the zero-order solution is given as:

9 $$\theta_{0} (t - \tau ) = \frac{1}{\sigma }\;\sin \,\sigma (t - \tau ).$$

The Homotopy Eq. (5) first-order problem can be expressed as:

10 $$\ddot{\theta }_{1} + \sigma^{2} \theta_{1} = \frac{1}{2}\omega^{2} \theta_{0}^{3} - \omega^{2} [\theta_{0}^{2} (t - \tau )\ddot{\theta } + \theta \dot{\theta }_{0}^{2} (t - \tau )],$$

according to the I. C.:

11 $$\theta_{1} (0) = 0,\,\,\,\,\dot{\theta }_{1} (0) = 0.$$

Usually, the secular terms are dropped to create a phrase that is consistently acceptable. The coefficients of the circular functions $\sin \sigma t$ and $\cos \sigma t$ would never be taken into consideration for this purpose. So, one realizes

12 $$3 + 8\sigma + 4\sigma^{2} (\cos^{2} \sigma \tau - \sin^{2} \sigma \tau ) = 0,$$

additionally with

13 $$2\tau - \sigma \cos \sigma \tau\,\sin\sigma \tau = 0.$$

Now, the first-order uniform formula is characterized as

14 $$\theta_{1} (t) = \frac{1}{{64\sigma^{3} }}\{ \sin 3\sigma t - 3\sin \sigma t\, + 4\sigma^{2} [( - 3\sin \sigma t + \sin 2\sigma t + \sin 3\sigma t)\cos 2\sigma \tau - \sin 2\sigma \tau \cos 3\sigma \tau ]\} .$$

Accordingly, the approximate uniform formula of the fundamental equation of movement described in Eq. (1) may be described as follows:

Consequently, the uniform estimated formula of Eq. (3) may be created as:

15 $$\theta (t) = \mathop {\lim }\limits_{\rho \to 1} e^{ - \delta \rho t/2} [\theta_{0} (t) + \rho \,\theta_{1} (t) + \cdots ].$$

Actuality, the uniform estimated formula in Eq. (15) needs that the functions should be of true significance. For this objective, combining Eqs. (7), (12), and (13), it is observed that the distinguishing nonlinear frequency verifies a specific equation. To this approximation, the computation has demonstrated that the nonlinear frequency validates:

16 $$48\sigma^{4} + 16(3 - 8\omega^{2} + 16\tau^{2} )\sigma^{2} + (3 - 8\omega^{2} )^{2} = 0.$$

Equation (16) represents the synthetic nonlinear characteristic frequency.

The approximated uniform solution, which is stated in Eq. (3), can be expressed as:

17 $$\theta = e^{ - \tau \,t} \left\{ {\frac{1}{\sigma }\;\sin \,\sigma + \frac{1}{16\sigma }[( - 3\sin \sigma t + \sin 2\sigma t + \sin 3\sigma t)\cos 2\sigma \tau - \sin 2\sigma \tau \cos 3\sigma \tau ]} \right\}.$$

**Figure 2:**
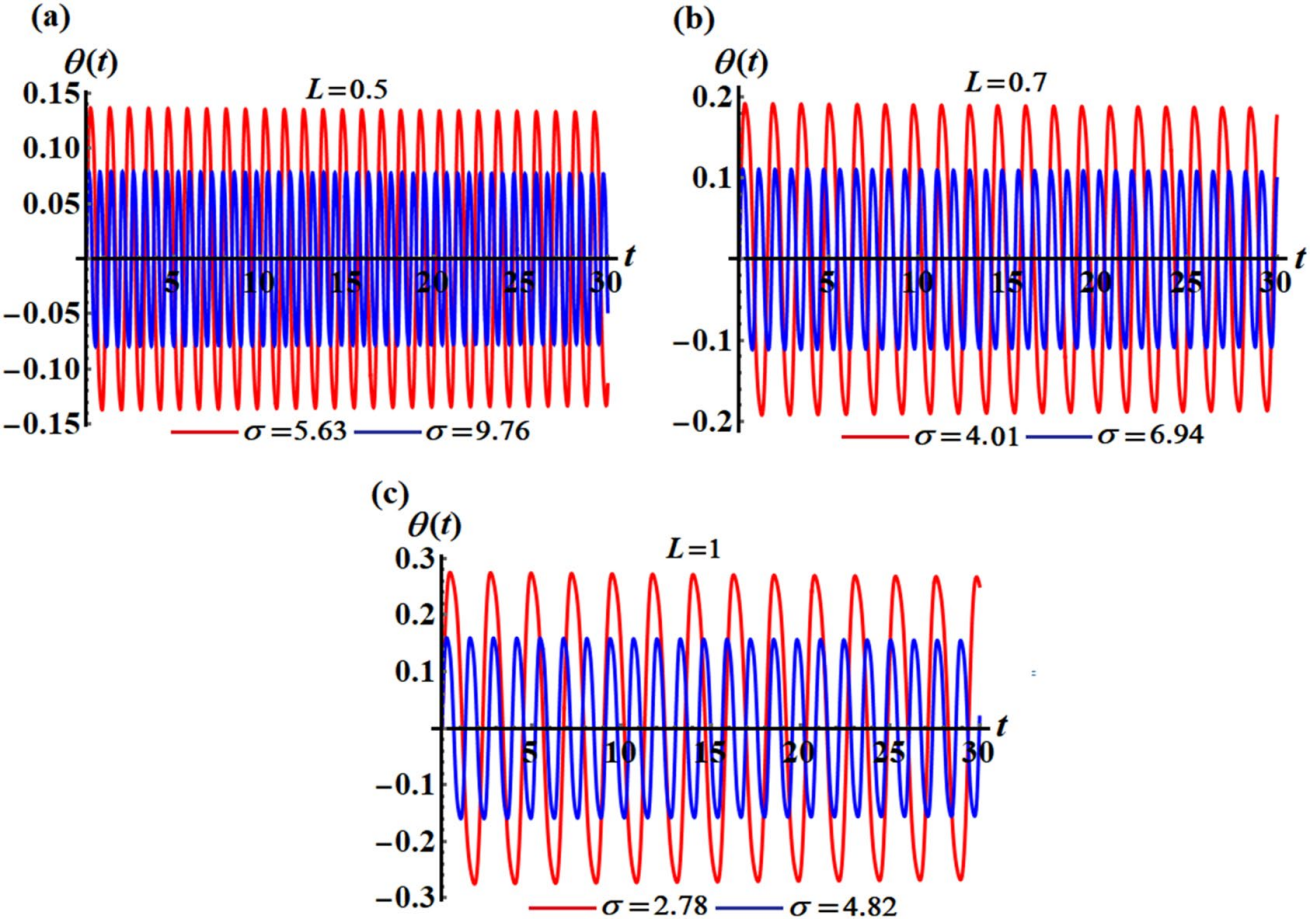
Represents the time dependent of the solution $\theta (t)$ at: (**a**) $L = 0.5,\sigma ( = 5.63,9.76)$, (**b**) $L = 0.7,\sigma ( = 4.01,6.94)$, and (**c**) $L = 1,\sigma ( = 2.78,4.82)$.

**Figure 3:**
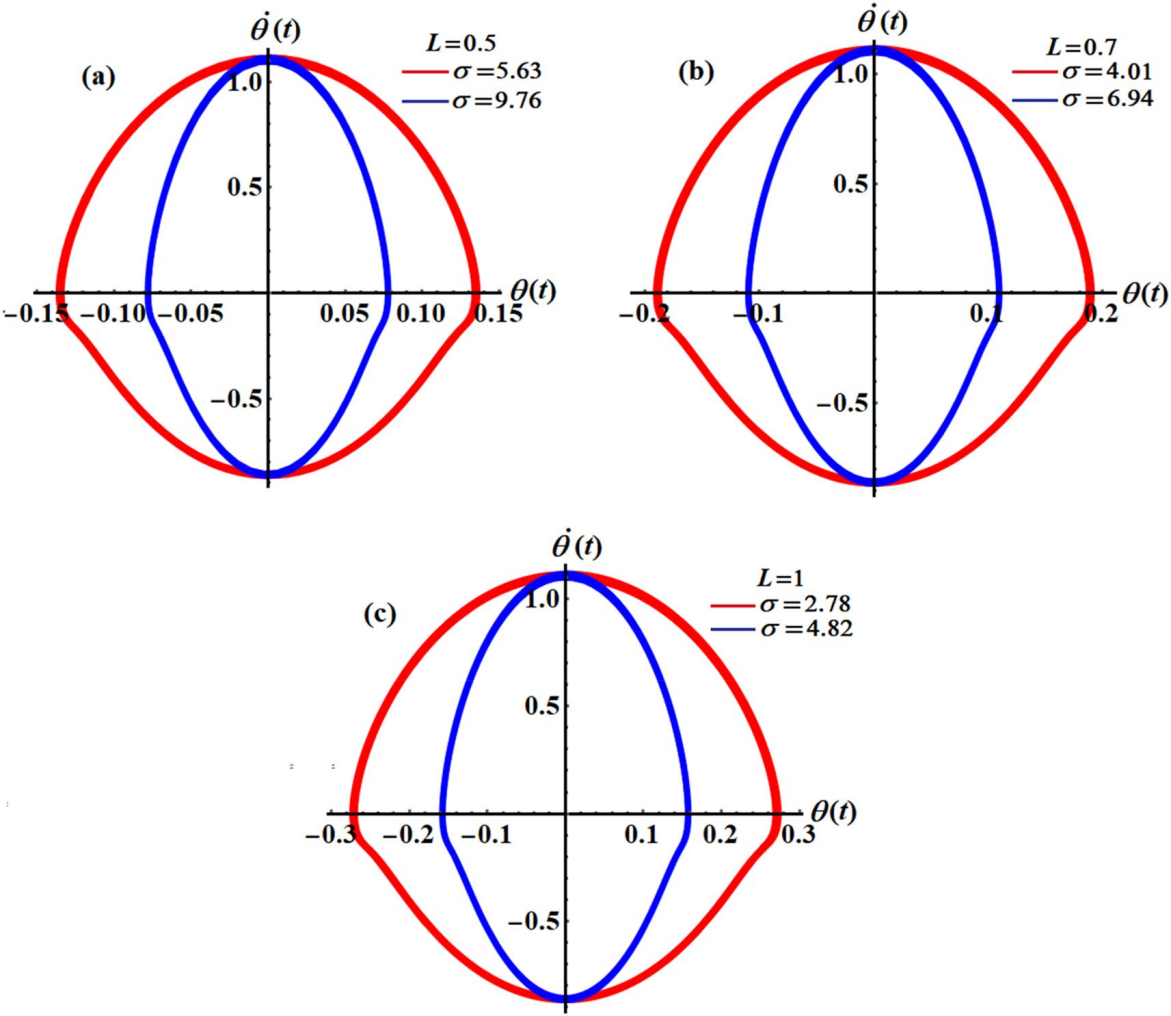
Portrays the corresponding curves of part of Fig. 2 in the plane $\theta \dot{\theta}$ at: (**a**) $L = 0.5,\sigma ( = 5.63,9.76)$, (**b**) $L = 0.7,\sigma ( = 4.01,6.94)$, and (**c**) $L = 1,\sigma ( = 2.78,4.82)$.

The above solution (17) has been drawn in Fig. 2 for various values of $L$ and $\sigma$, where portions (a), (b), and (c) are graphed at $(L = 0.5,\sigma = 5.63,9.76),$ $(L = 0.7,\sigma = 4.01,6.94),$ and $(L = 1,\sigma = 2.78,4.82),$ respectively. It should be noted that, in order to obtain a good precision, the Mathematica Software Version 12.0.0.0 will be used in the following numerical computations. It is noted that the plotted curves have uniform periodic forms, which assert that the obtained solution has a stable manner. Moreover, when $L$ rises from $0.5$ to $1$ to the value $0.7$, the amplitudes of the represented waves increase while the oscillation number decrease. Moreover, when the value of $\sigma$ increases (for the same value of $L$), we can observe that the amplitude of the repeated waves decreases while the oscillation number increases. It is important to observe that the depicted curves line up with the mathematical formulas of Eq. (17). Curves of the phase plane diagrams in parts of Fig. 2 are drawn in the corresponding parts of Fig. 3 to assert the stability of the gained solution. These curves have the forms of closed curves and they are plotted in the plane $\theta \dot{\theta }$, where $\dot{\theta }$ represent the first derivative of the solution (17) with time. Therefore, one can say that these curves illustrate that the solution has a stable form.

It is suitable to evaluate this procedure with the numerical solution (NS), which can be obtained using the computational approach that is identified to RK4 to evaluate the practicality of the formerly expanded frequency implications. The requirements for implementation are listed below. Therefore, the analytical solution (AS) as given by Eq. (17) is drawn in blue. Additionally, the RK4 of the problem under consideration, as provided by Eq. (3), is highlighted in red. The subsequent graph is a graph of a structure receiving the specifics:

$$\,L = 1.0,\,\,\,\,{\text{and}}\,\,\,\tau = 0.001.$$

**Figure 4:**
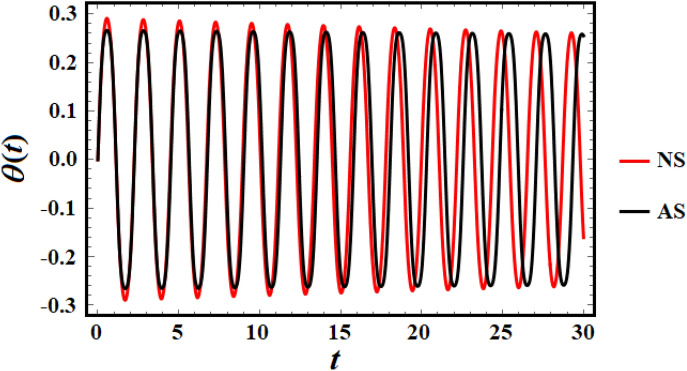
Perturbed and numerical solutions of Eq. (27).

The computations demonstrated that the synthetic frequency has an amount $\sigma = 2.78388$ and other roots (two are complex conjugates and the third is negative). It is clear from Fig. 4 how close the two solutions are to each other, which in turn highlights the accuracy of the perturbation method used. This reveals that expanded frequency, as an estimated formula, is a favorable and effective perturbation procedure.

**Figure 5:**
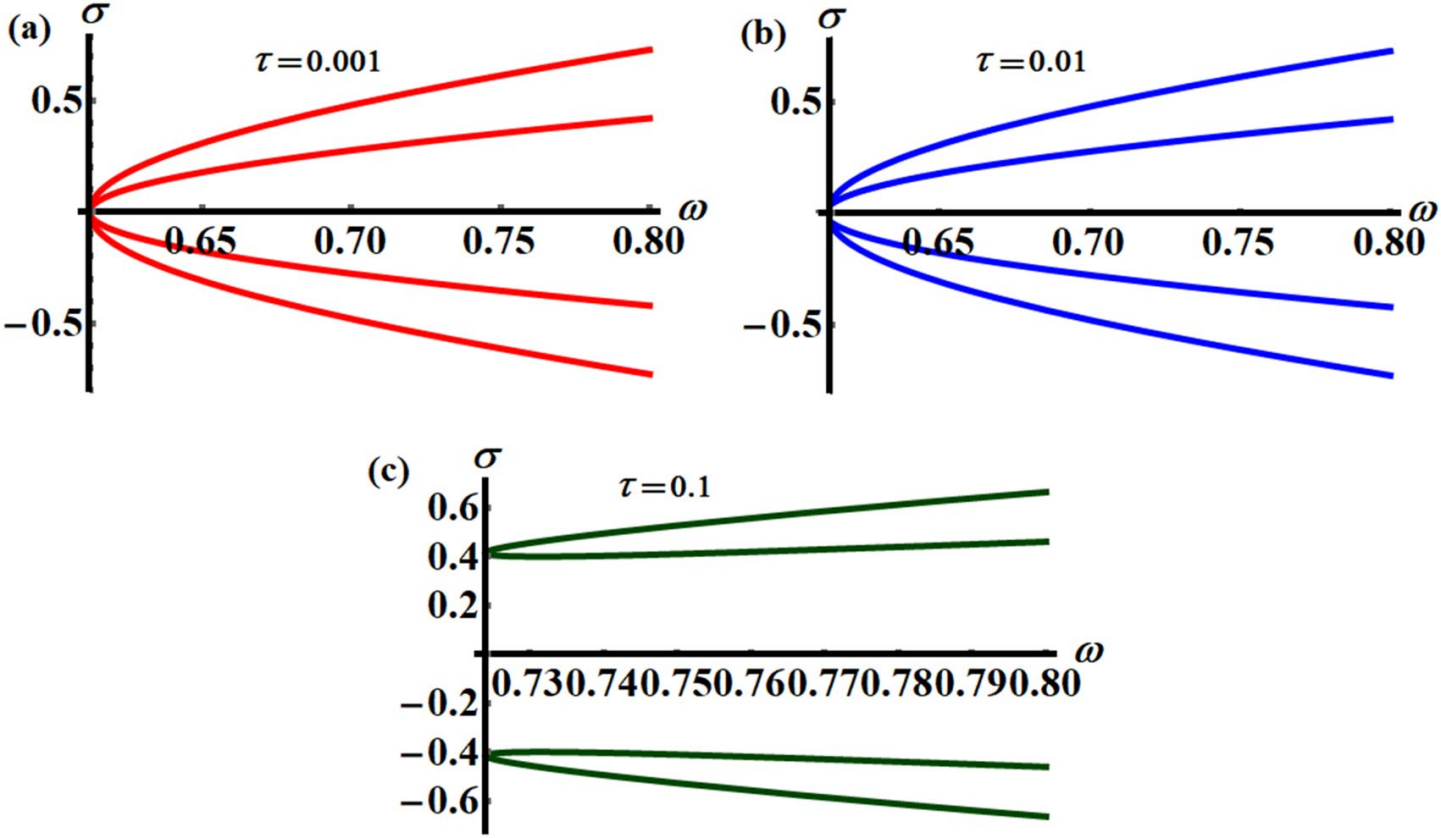
Reveals the variation of $\omega$ with $\sigma$ according to different values of time delay: (**a**) $\tau = 0.001$, (**b**) $\tau = 0.01$, and (**c**) $\tau = 0.1$.

Equation (16) of the fourth degree of $\sigma$ has been plotted versus $\omega$, as seen in Fig. 5, at various values of the time delay parameter $\tau ( = 0.001,0.01,0.1)$. It deserves to be highlighted that the calculated curves in portions (a), (b), and (c) have fork forms, where they are symmetric about the $\omega$-axis. According to the presented results, the amplitude between the two branches of the fork decreases with the increase of $\tau$ values, where we find that the distance between the original point of $\sigma$ and the $\omega$ axes, and the initial points of the drawn fork curve diverge. The reason goes back to the four roots of Eq. (16), in which two of them are drawn in the first quartile**,** while the others are graphed in the fourth quartile. Consequently, there is symmetry in the curves drawn around the $\omega$-axis.

## Linearized stability

The purpose here is to depict the stability analysis as well as the phase portrait of the EOM in the absence of the time delay as given in Eq. (2). For this objective, the ODE can be converted into a structure of two first-order ones. This can be accomplished through the transformation $\dot{\theta } = \varphi$. Therefore, the resulting system may be displayed as:

18 $$\dot{\theta } = g(\theta ,\,\,\varphi ),\,\,\,\,\dot{\varphi } = h(\theta ,\,\varphi ),$$

where

19 $$g(\theta ,\,\varphi ) = \varphi ,\,\,\,\,\,\,\,\,h(\theta ,\,\,\varphi ) = - \frac{\theta }{{2(1 + \omega^{2} \theta^{2} )}}(2 - \theta^{2} + 2\varphi^{2}) \omega^{2}.$$

The equilibrium points happen at the points $(\theta_{0} ,\,\varphi_{0} )$, where

20 $$\begin{array}{*{20}c} {\varphi_{0} = 0,} \\ {2\theta_{0} - \theta_{0}^{3} + 2\theta_{0} \varphi_{0}^{2} = 0.} \\ \end{array}$$

It follows that the fixed points are given at $(0,\,0)$ and $( \pm \sqrt 2 ,\,0)$.

In considering the Taylor series, the functions $g(\theta ,\,\varphi )$ and $h(\theta ,\,\varphi )$ will be expanded around the fixed point. Taking merely the linear terms, one reaches the Jacobian formula:

21 $$J = \left( {\begin{array}{*{20}c} 0 & 1 \\ {\frac{{ - 2(1 + \varphi_{0}^{2} ) + \theta_{0}^{2} [3 + (2 + \theta_{0}^{2} + 2\varphi_{0}^{2} )]}}{{2(1 + \omega^{2} \theta_{0}^{2} )^{2} }}\omega^{2} } & { - \frac{{2\omega^{2} \theta_{0} \varphi_{0} }}{{(1 + \omega^{2} \theta_{0}^{2} )}}} \\ \end{array} } \right).$$

As previously shown^3^, the eigenvalues $\Lambda$ for this structure is defined as follows:

22 $$\left| {\begin{array}{*{20}c} { - \Lambda } & 1 \\ {\frac{{ - 2(1 + \varphi_{0}^{2} ) + \theta_{0}^{2} [3 + (2 + \theta_{0}^{2} + 2\varphi_{0}^{2} )]}}{{2(1 + \omega^{2} \theta_{0}^{2} )^{2} }}\omega^{2} } & { - \frac{{2\omega^{2} \theta_{0} \varphi_{0} }}{{(1 + \omega^{2} \theta_{0}^{2} )}} - \Lambda } \\ \end{array} } \right| = 0.$$

The equilibrium point is traditionally considered to be stable if all eigenvalues, which are computed at the equilibrium points, have negative real portions. As previously shown^3^, the final classification can be used for this methodology, as seen in Table 1.

**Table 1:** Demonstrates the different eigenvalue categories.

Example of selected scheme	Fixed point	Roots of the eigenvalues	Stability/instability
$L = 1$	$(0,\,0)$	Pure imaginary $\Lambda_{1,2} = \pm 3.4\,i$	A stable center See Fig. 6
	$( \pm \sqrt 2 ,\,0)$	Real, equal, and different sign $\Lambda_{1,2} = \pm 0.98$	
$L = \sqrt {12}$	$(0,\,0)$	Pure imaginary $\Lambda_{1,2} = \pm \,i$	A stable center See Fig. 7
	$( \pm \sqrt 2 ,\,0)$	Real, equal, and different sign $\Lambda_{1,2} = \pm 0.82$

**Figure 6:**
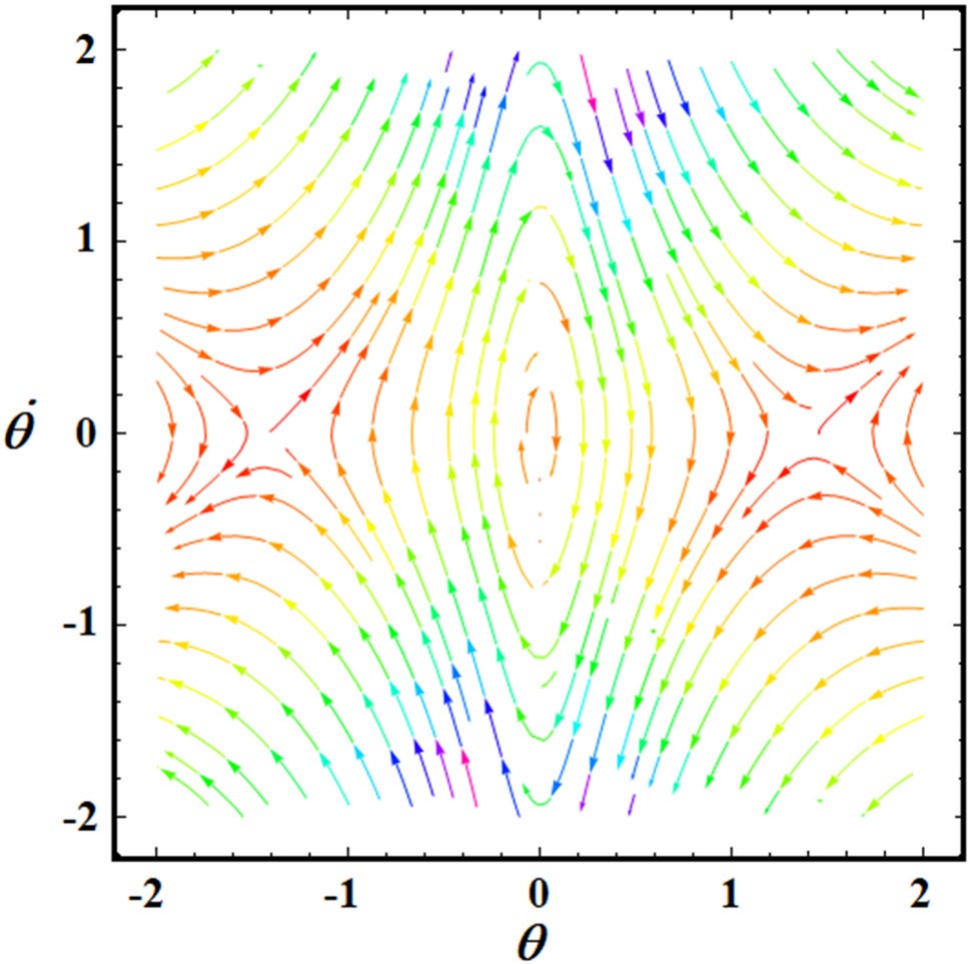
Depicts the phase portrait at $L = 1$.

**Figure 7:**
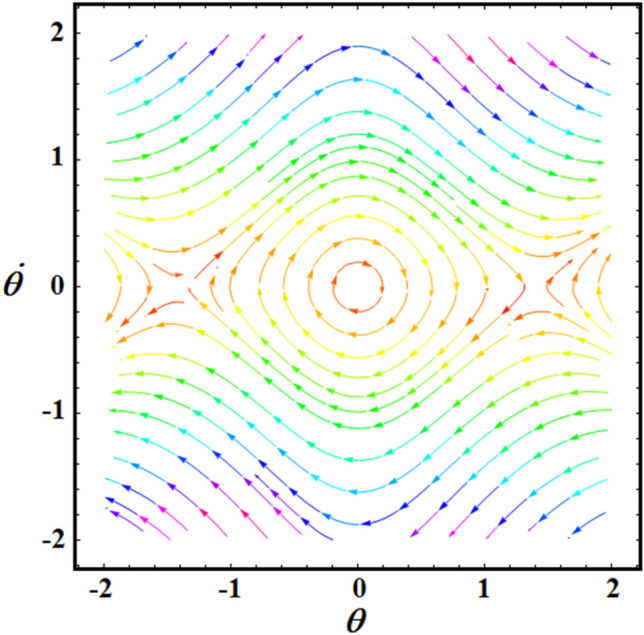
Depicts the phase portrait at $L = \sqrt {12}$.

## Multiple-time scales method

This method is used to calculate the stability behavior of Eq. (5) for various times scales^29^. One might also think of the explanatory variables as a function of $t$ given the HPM. As a result, rather than treating the enlargement a consequence of only one independent variable, or scale, it is handled as a function of several time scales. The perturbation theory actually views the multiple-time scales procedure a more generic approach.

For this objective, one starts by presenting three different independently variable quantities corresponding to:

23 $$T_{n} = \rho^{n} t, n = 0,1, \ldots$$

Consequently, one gets:

24 $$\frac{d}{dt} \equiv \frac{{dT_{0} }}{dt}\frac{\partial }{{\partial T_{0} }} + \frac{{dT_{1} }}{dt}\frac{\partial }{{\partial T_{1} }} + \cdots = D_{0} + \rho D_{1} + \rho^{2} D_{2} + \cdots ,$$

and

25 $$\frac{{d^{2} }}{{dt^{2} }} \equiv D_{0}^{2} + 2\rho D_{0} D_{1} + \rho^{2} (D_{1}^{2} + 2D_{0} D_{2} ) + \cdots ,$$

where $$D_{n} \equiv \frac{\partial }{{\partial T_{n} }}.$$

The solution of Eq. (5) may be characterized as an extension in the subsequent structure:

26 $$\theta (t;\rho ) = \theta_{0} (T_{0} ,T_{1} ,T_{2} , \ldots ) + \rho \theta_{1} (T_{0} ,T_{1} ,T_{2} , \ldots ) + \rho^{2} \theta_{2} (T_{0} ,T_{1} ,T_{2} , \ldots ) + \cdots$$

In view of the Homotopy perturbation, Eq. (2) might be represented as:

27 $$\ddot{\theta }(t) + \omega^{2} \theta (t) + \rho \{ \omega^{2} [\theta^{2} (t - \tau )\ddot{\theta }(t) + \theta (t) \dot{\theta }^{2} (t - \tau )] - \frac{{\omega^{2} }}{2}\theta^{3} (t)\} = 0; \rho \in [0,1].$$

For more accessibility, to achieve a precise formula, we restrict our analysis up to $O(\rho )$. In this situation, only one-time scales, $T_{0} ,T_{1} ,$ and $T_{2}$ are needed.

Substituting from Eqs. (24)–(26) into Eq. (27), one gets

28 $$\rho^{0} : (D_{0}^{2} + \omega^{2} )\theta_{0} = 0,$$

29 $$\begin{aligned} \rho : (D_{0}^{2} + \omega^{2} )\theta_{1} & = 2D_{0} D_{1} \theta_{0} (T_{0} ,T_{1} ,T_{2} ) + \omega^{2} \{ \theta_{0}^{2} (T_{0} - \tau ,T_{1} ,T_{2} )D_{0}^{2} \theta_{0} (T_{0} ,T_{1} ,T_{2} ) \\ & \quad + \theta_{0} (T_{0} ,T_{1} ,T_{2} )[D_{0} \theta_{0} (T_{0} - \tau ,T_{1} ,T_{2} )]^{2} - \frac{{\omega^{2} }}{2}\theta_{0}^{3} (T_{0} ,T_{1} ,T_{2} )\} , \\ \end{aligned}$$

and

30 $$\begin{aligned} \rho^{2} : (D_{0}^{2} + \omega^{2} )\theta_{2} & = - \{ D_{1}^{2} \theta_{0} (T_{0} ,T_{1} ,T_{2} ) + 2D_{0} D_{2} \theta_{0} (T_{0} ,T_{1} ,T_{2} ) + 2D_{0} D_{1} \theta_{1} (T_{0} ,T_{1} ,T_{2} ) \\ & \quad - \frac{{3\omega^{2} }}{2}\theta_{1} (T_{0} ,T_{1} ,T_{2} ) \theta_{0}^{2} (T_{0} ,T_{1} ,T_{2} ) + 2\omega^{2} \theta_{0} (T_{0} ,T_{1} ,T_{2} )D_{1} \theta_{0} (T_{0} - \tau ,T_{1} ,T_{2} ) \\ & \quad \times D_{0} \theta_{0} (T_{0} - \tau ,T_{1} ,T_{2} ) + \omega^{2} \theta_{1} (T_{0} ,T_{1} ,T_{2} ) [D_{0} \theta_{0} (T_{0} - \tau ,T_{1} ,T_{2} )]^{2} \\ & \quad + 2\omega^{2} \theta_{0} (T_{0} ,T_{1} ,T_{2} )D_{0} \theta_{0} (T_{0} - \tau ,T_{1} ,T_{2} )D_{0} \theta_{1} (T_{0} - \tau ,T_{1} ,T_{2} ) \\ & \quad + 2\omega^{2} \theta_{0}^{2} (T_{0} - \tau ,T_{1} ,T_{2} )D_{0} D_{1} \theta_{0} (T_{0} ,T_{1} ,T_{2} ) + 2\omega^{2} \theta_{0} (T_{0} - \tau ,T_{1} ,T_{2} ) \\ & \quad \times \theta_{1} (T_{0} - \tau ,T_{1} ,T_{2} )D_{0}^{2} \theta_{0} (T_{0} ,T_{1} ,T_{2} ) + \omega^{2} \theta_{0}^{2} (T_{0} - \tau ,T_{1} ,T_{2} )D_{0}^{2} \theta_{1} (T_{0} ,T_{1} ,T_{2} ) \} . \\ \end{aligned}$$

With this methodology, it is appropriate to create the solution of Eq. (28) in the following form:

31 $$\theta_{0} (T_{0} ,T_{1} ,T_{2} ) = A(T_{1} ,T_{2} )e^{{i\omega T_{0} }} + \overline{A}(T_{1} ,T_{2} )e^{{ - i\omega T_{0} }} .$$

Here, $A$ is an unspecified complex function that can be established later on, and $\overline{A}$ is a corresponding complex conjugate.

Substituting Eq. (31) into Eq. (29), one finds

32 $$\begin{aligned} (D_{0}^{2} + \omega^{2} )\theta_{1} & = \frac{1}{2}\omega^{2} A^{3} e^{{3i\omega T_{0} }} (1 + 4e^{ - 2i\tau \omega } \omega^{2} ) + \frac{1}{2}\omega^{2} \overline{A}^{3} e^{{ - 3i\omega T_{0} }} (1 + 4e^{2i\tau \omega } \omega^{2} ) + e^{{i\omega T_{0} }} [\frac{1}{2}\omega^{2} A^{2} \overline{A} \\ & \quad \times (3 + 4e^{ - 2i\tau \omega } \omega^{2} ) - 2i\omega D_{1} A] + {\text{e}}^{{ - i\omega T_{0} }} [\frac{1}{2}\omega^{2} A\overline{A}^{2} (3 + 4e^{2i\tau \omega } \omega^{2} ) + 2i\omega D_{1} \overline{A}]. \\ \end{aligned}$$

The removal of the undesired parts, in Eq. (32), at the non-resonant case gives

33 $$\omega^{2} A^{2} \overline{A}(3 + 4e^{ - 2i\tau \omega } \omega^{2} ) - 4i\omega D_{1} A = 0.$$

It worth observing that the consistently reasonable formula of Eq. (32) can be represented as

34 $$\theta_{1} (T_{0} ,T_{1} ,T_{2} ) = - \frac{1}{16}[A^{3} {\text{e}}^{{3i\omega T_{0} }} (1 + 4e^{ - 2i\tau \omega } \omega^{2} ) + \overline{A}^{3} e^{{ - 3i\omega T_{0} }} (1 + 4e^{2i\tau \omega } \omega^{2} )].$$

Again, the exclusion of the unsought pieces, in Eq. (30), at the non-resonant case gives

35 $$\begin{aligned} & \frac{1}{8}e^{ - 2i\tau \omega } \omega^{4} A^{3} \overline{A}^{2} (1 - 8 e^{ - 2i\tau \omega } \omega^{2} + 5e^{4i\tau \omega } + 20e^{i\tau \omega } \omega^{2} ) + \frac{3}{32}\omega^{2} A^{3} \overline{A}^{2} \\ & \quad + 2i\omega^{3} A\overline{A}D_{1} A(1 + e^{ - 2i\tau \omega } ) + 2i\omega^{3} A^{2} D_{1} \overline{A}(1 - e^{ - 2i\tau \omega } ) + D_{1}^{2} A + 2i\omega D_{2} A = 0. \\ \end{aligned}$$

Equation (34) may be simplified by removing the terms $D_{1}^{2} A,D_{1} A,$ and $D_{1} \overline{A}$ from the solvability criterion of the first non-resonance argument as offered by Eq. (33), to find

36 $$\begin{aligned} & e^{ - 4i\tau \omega } \omega^{2} [32\omega^{4} + 4{\text{e}}^{6i\tau \omega } \omega^{2} (16\omega^{2} - 11) - 4e^{2i\tau \omega } \omega^{2} (16\omega^{2} + 7) \\ & \quad + e^{4i\tau \omega } (15 - 176\omega^{4} )]A^{3} \overline{A}^{2} - 64i\omega D_{2} A = 0. \\ \end{aligned}$$

To go back to the initial varying $t$, we may reproduce Eq. (33) with the factor $\rho$, and proliferate Eq. (36) by $\rho^{2}$, then add up them as one. Ultimately, taking up the limit as $\rho \to 1$, one realizes

37 $$\begin{aligned} & 16\omega (3 + 4e^{ - 2i\tau \omega } \omega^{2} )A^{2} \overline{A} + e^{ - 4i\tau \omega } \omega [32\omega^{4} + 4e^{6i\tau \omega } \omega^{2} (16\omega^{2} - 11) - 4e^{2i\tau \omega } \omega^{2} (16\omega^{2} + 7) \\ & \quad + e^{4i\tau \omega } (15 - 176\omega^{4} )]A^{3} \overline{A}^{2} - 64i\frac{dA}{{dt}} = 0. \\ \end{aligned}$$

As usual, the solution of Eq. (36) may be formulated as:

38 $$A(t) = \alpha (t)e^{i\beta (t)} ,$$

where $\alpha (t)$ and $\beta (t)$ are real functions.

Combining Eqs. (38) and (37), one finds

39 $$\alpha^{\prime}(t) = 0,$$

and

40 $$\begin{aligned} & 16\omega (3 + 4e^{ - 2i\tau \omega } \omega^{2} )\alpha^{2} (t) + e^{ - 4i\tau \omega } \omega [32\omega^{4} + 4e^{6i\tau \omega } \omega^{2} (16\omega^{2} - 11) - 4e^{2i\tau \omega } \omega^{2} (16\omega^{2} + 7) \\ & \quad + e^{4i\tau \omega } (15 - 176\omega^{4} )]\alpha^{4}(t) + 64\beta^{\prime}(t) = 0. \\ \end{aligned}$$

Solving Eqs. (35) and (36), one finds $\alpha = c_{1}$ and

41 $$\begin{aligned} \beta (t) & = c_{2} + \frac{1}{64}c_{1}^{2} \omega \,t\{ - 48 - 32{\text{e}}^{ - 4i\tau \omega } c_{1}^{2} \omega^{4} - 4{\text{e}}^{2i\tau \omega } c_{1}^{2} \omega^{2} (16\omega^{2} - 11) + c_{1}^{2} (176\omega^{4} - 15) \\ & \quad + 4{\text{e}}^{ - 2i\tau \omega } \omega^{2} [c_{1}^{2} (16\omega^{2} + 7) - 16]\} , \\ \end{aligned}$$

where $c_{2}$ is an integration constant.

**Figure 8:**
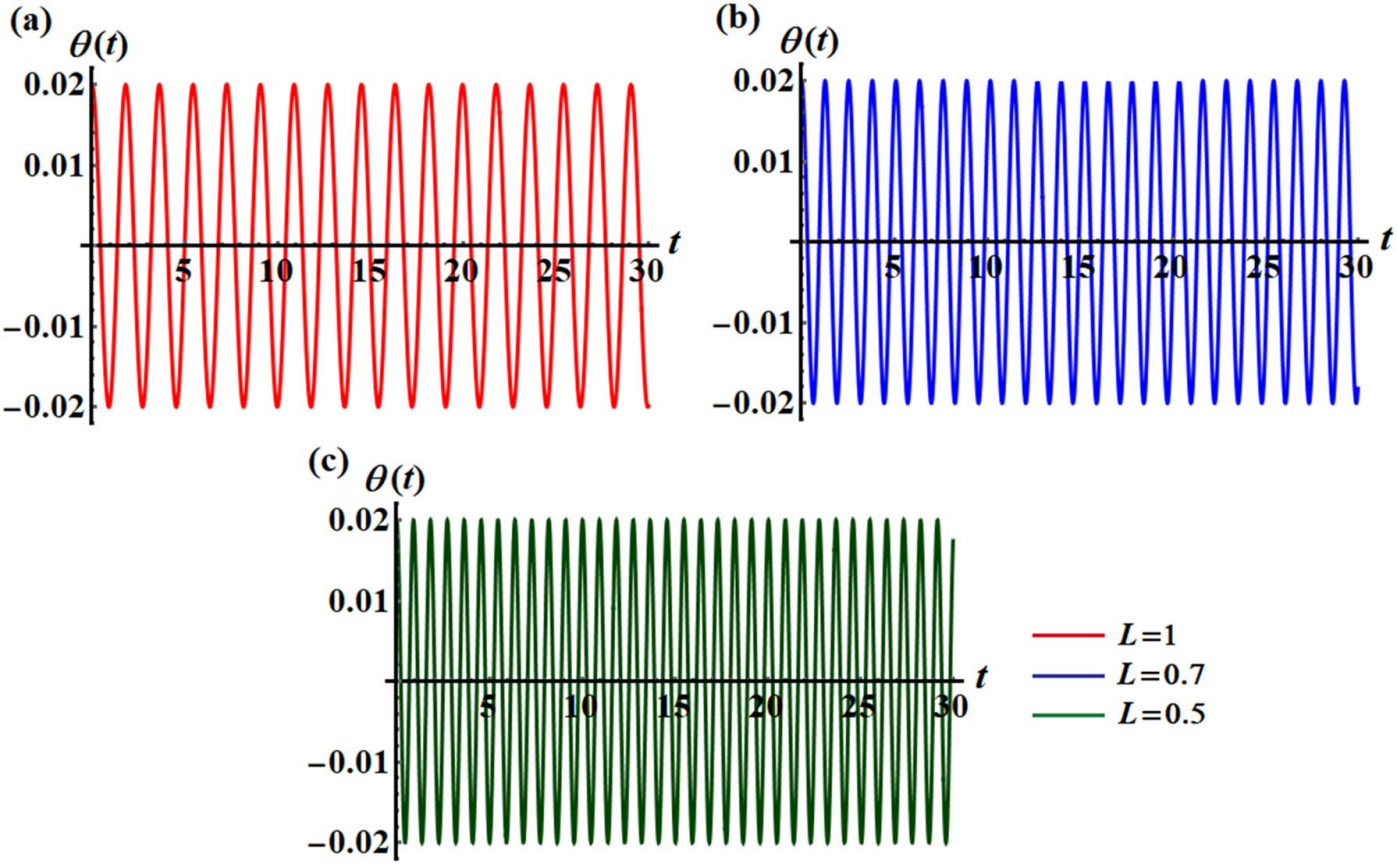
Describes the temporaly history of $\theta$ when $\tau = 0.0$ at: (**a**) $L = 0.5,\sigma = 2.78$, (**b**) $L = 0.7,\sigma = 4.01$ (**c**) $L = 1.0,\sigma = 5.63$.

**Figure 9:**
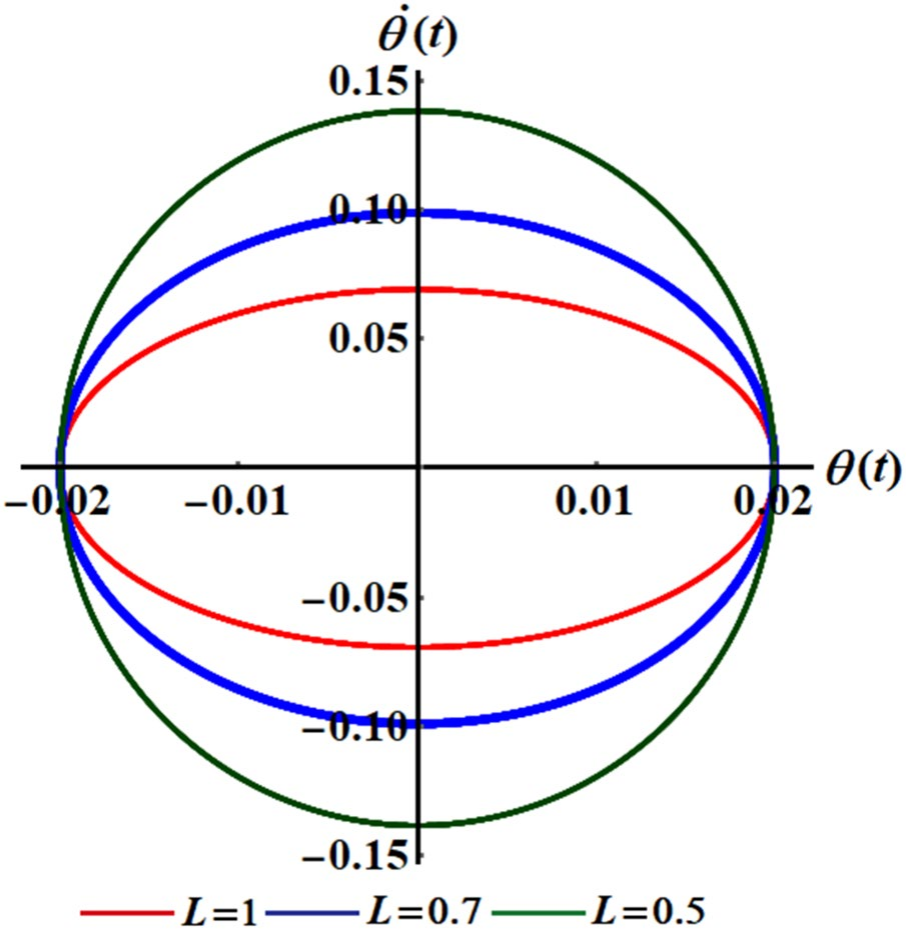
Reveals the curves of Fig. 8 in the plane $\theta \dot{\theta }$.

Curves of Fig. 8 show the time history of the obtained findings by the multiple-time scale method, and they are drawn at various values of $L( = 1.0,0.7,0.5)$ when $\sigma ( = 2.78,4.01,5.63)$. These curves have a uniform periodic behavior, in which the numeral of vibrations rises while the wave numbers decrease with the decrease of the amounts of $L$, as seen in portions (a), (b), and (c). Therefore, the motion is stable and free of chaos. To assert this statement, the phase plane diagram for the same amounts of these curves in Fig. 8 is plotted in Fig. 9 to draw closed symmetric curves which correspond to the curves of Fig. 8.

**Figure 10:**
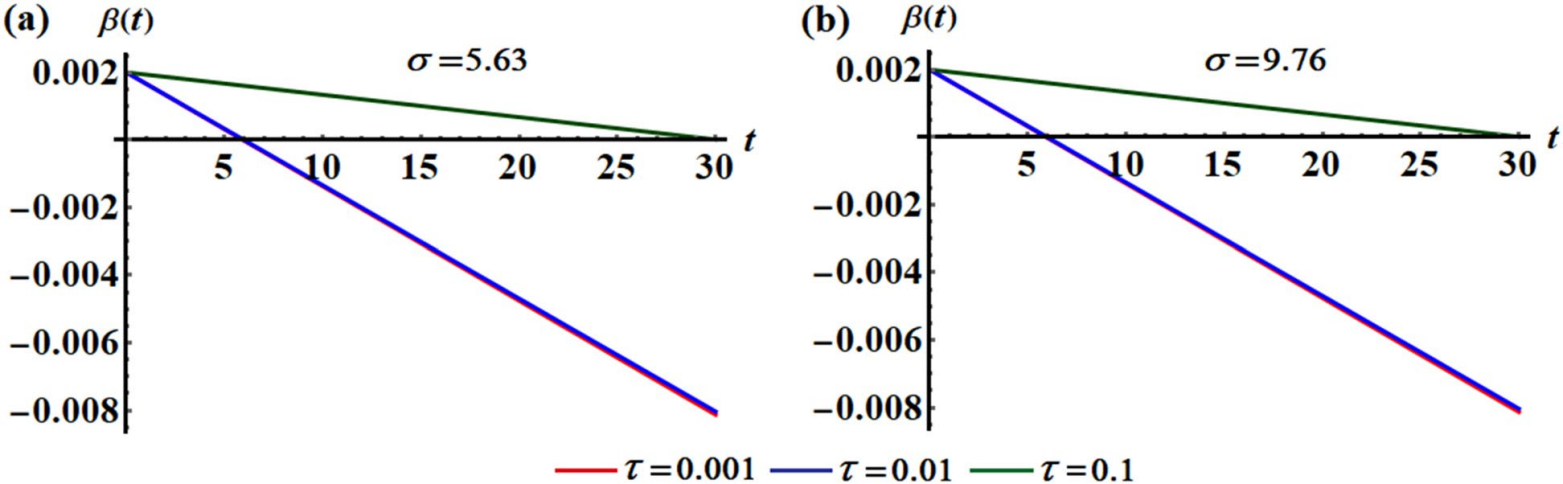
Shows the temporaly histories of $\beta$ when $L = 0.5$ and $\tau ( = 0.001,0.01,0.1)$ at: (**a**) $\sigma = 5.63$, (**b**) $\sigma = 9.76$.

**Figure 11:**
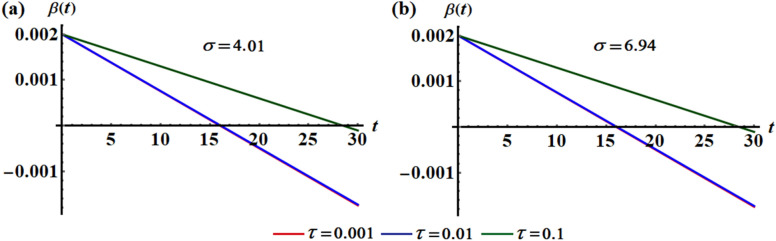
Shows the temporaly histories of $\beta$ when $L = 0.7$ and $\tau ( = 0.001,0.01,0.1)$ at: (**a**) $\sigma = 4.01$, (**b**) $\sigma = 6.94$.

The variation of the function $\beta$ via time at various values of the time delay parameter $\tau ( = 0.001,0.01,0.1)$ is plotted in Figs. 10 and 11. Parts (a) and (b) of Fig. 10 are calculated at $L = 0.5$ when $\sigma = 5.63$ and $\sigma = 9.76$, respectively. Additionally, curves of Fig. 11 are calculated at $L = 0.7$ when $\sigma = 4.01$ and $\sigma = 6.94$. According to the previous equation, one can expect that the graphed curves in these figures have straight lines behavior, in which they start from one initial point and have a decay form according to the values of the decaying parameter. It is noted that the angle between the lines of each figure decreases with the increase of $\sigma$ amounts, as graphed in portions (a) and (b) of Figs. 10 and 11, while this angle decreases as $L$ increases, as noticed in the corresponding parts of these graphs. This means that the time delay factor has a positive impact on the behavior of function $\beta$.

## Concluding remarks

The present investigation looks at the subject of an inflexible rod revolving on a solid rod as a spherical surface with no sliding. In our previous study^10^, the EOM was developed on the basis of the Euler–Lagrange theorem. This work applies a time-delayed square position as well as speed to examine the stability analysis of a rocking rigid rod in line with the perception of time delay. The subject of this inquiry is extremely significant since time-delayed technology has recently been the subject of several investigations. A coupling of the enlarged nonlinear frequency notion and the HPM is employed in view of the time delay. This investigation is significant since the time-delayed technology issue of this investigation is extremely relevant. An exponential term is also introduced to obtain a rough solution of the EOM in light of the time delayed factor. The temporally histories of the obtained solution have been plotted at various values of the used parameters. It is noted that the behavior of graphed curves has a periodic form, in which the phase plane plots of the obtained outcomes assert their stabilities. To validate the accuracy of the theoretical outcome, a comparison with the RK4 is provided. The present study allows a careful analysis of the conclusions reached using the analytical strategy of practical approximation. The time delay acts as an extra protection against the system potential for nonlinear oscillation. In contrast to earlier research, the methodology arrived at the current answer is notable for being effective, promising, and simple. The multiple scale method is used to test if it can be applied to additional nonlinear oscillators even with the organized nonlinear prototype approach. It has investigated how various regulatory constraints affected foundation vibration achievements.

Another non-perturbation procedure built on the well-known He's frequency^30–33^ is suitable for use as a prototype for the latest research. With this methodology, the nonlinear EOM as shown in Eq. (1) is transformed into a linear one. The novel method appears effective and interesting and can be used in the study from different classes of nonlinear partial/ordinary differential equations. Additionally, the stability requirements of the nonlinear differential equation can also be easily met.

## Data Availability

All data generated or analysed during this study are included in this published article.

## List of symbols

$\theta$: Angular movement at any time
$$t$$: Proper duration
$^{.}$: Derivative with respect to time is symbolized by a point
$\omega$: Regular frequency of the structure
$\tau$: Time-decay control
$a$: Dimensional length of the rocking rod
$$g$$: Gravitational acceleration
$r$: Radius of the circular surface
$M$: Mass of the uniform rigid rod
$C.M.$: Center of mass of the consistently inflexible rod
$L$: Non-dimensional length of the rocking rod
$\delta ,\,\,\rho$: Slight synthetic constraints
